# Epileptic Disorder Detection of Seizures Using EEG Signals

**DOI:** 10.3390/s22176592

**Published:** 2022-08-31

**Authors:** Mariam K. Alharthi, Kawthar M. Moria, Daniyal M. Alghazzawi, Haythum O. Tayeb

**Affiliations:** 1 Department of Computer Science, College of Computing and Information Technology, King Abdulaziz University, Jeddah 21589, Saudi Arabia; 2 Department of Information Systems, College of Computing and Information Technology, King Abdulaziz University, Jeddah 21589, Saudi Arabia; 3 The Neuroscience Research Unit, Faculty of Medicine, King Abdulaziz University, Jeddah 21589, Saudi Arabia

**Keywords:** CHB-MIT dataset, deep learning, epilepsy, seizure detection, XLtek EEG

## Abstract

Epilepsy is a nervous system disorder. Encephalography (EEG) is a generally utilized clinical approach for recording electrical activity in the brain. Although there are a number of datasets available, most of them are imbalanced due to the presence of fewer epileptic EEG signals compared with non-epileptic EEG signals. This research aims to study the possibility of integrating local EEG signals from an epilepsy center in King Abdulaziz University hospital into the CHB-MIT dataset by applying a new compatibility framework for data integration. The framework comprises multiple functions, which include dominant channel selection followed by the implementation of a novel algorithm for reading XLtek EEG data. The resulting integrated datasets, which contain selective channels, are tested and evaluated using a deep-learning model of 1D-CNN, Bi-LSTM, and attention. The results achieved up to 96.87% accuracy, 96.98% precision, and 96.85% sensitivity, outperforming the other latest systems that have a larger number of EEG channels.

## 1. Introduction

Epilepsy is a neurological disorder that affects children and adults. It can be characterized by sudden recurrent epileptic seizures [1]. This seizure disorder is basically a temporary, brief disturbance in the electrical activity of a set of brain cells [2]. The excessive electrical activity inside the networks of neurons in the brain will cause epileptic seizures [3]. These seizures result in involuntary movements that may include part of the body (partial movement) or the whole body (generalized movement) and are sometimes accompanied by disturbances of sensation (involving hearing, vision, and taste), cognitive functions, mood, or may cause loss of consciousness [2]. The frequency of seizures varies from patient to patient, ranging from less than once a year to several times a day. Active epilepsy patients have a mortality proportion of 4–5 times greater than seizure-free people [4]. However, effective medical therapy that is individualized for each individual patient helps to lower the risk of mortality. Reduced mortality can be achieved by objectively quantifying both seizures and the response to therapy [5].

The seizure detection modality uses an electroencephalogram (EEG) [6]. Signals monitor the brain’s electrical activity through electrodes. An electrode is a small metal disc that attaches to the scalp to capture the brainwave activity through the EEG channel, which, depending upon the EEG recording system, can range from 1 channel to 256 channels. EEG signals are in the form of sinusoidal waves with different frequencies that neurophysiologists use to identify brain abnormalities. One major challenge that neurologists face is the presence of EEG signal artifacts. EEG signals overlapped with other internal and external bio-signals cause artifacts that mimic the EEG seizure signal and thus give false data. Some examples include eye movement, cardiogenic movement, muscle movement, or environmental noise [7]. Table 1 illustrates the frequency bands of EEG signals with normal and abnormal tasks affecting each band. Neurophysiologists need to collect an extensive amount of long-term EEG signals in order to detect seizures through visual analysis of these signals in a time-consuming manual process.

There is a current, urgent need to develop a generalized automatic seizure detection system that provides precise seizure quantification, allowing neurophysiologists to objectively tailor treatment. Developing such a system is challenging because the available datasets are mostly imbalanced; the number of non-seizure EEG signals is larger than the number of EEG seizure signals in the datasets [9]. This imbalanced dataset issue can have a major negative impact on classification performance [10].

This research proposes a compatibility framework to integrate local EEG data from an epilepsy center at King Abdulaziz University hospital (KAU) with the CHB-MIT dataset [11] to solve the problem of limited resources and imbalanced data. It also proposes an algorithm for reading XLtek EEG data, incorporated into the proposed framework, thus allowing researchers to analyze this type of EEG signal for which no auxiliary analytical tools are available in the dedicated packages. Finally, a deep-learning seizure-detection model based on selected EEG channels has been developed. The results show that the proposed method outperforms other models that rely on using a larger number of EEG channels to detect epileptic seizures.

The CHB-MIT dataset was chosen as it has the same type of scalp EEG recordings and annotations as the KAU local dataset. Additionally, the CHB-MIT has recordings from all parts of the brain that contain similar seizure types as those in the KAU dataset, such as clonic, tonic, and atonic seizures.

The rest of the paper is organized as follows: Section 2 presents the state-of-the-art seizure detection systems. In Section 3, the datasets that were used in the research are described. Section 4 explains the proposed approaches. The evaluation of each approach over the CHB-MIT benchmark EEG dataset with the KAU dataset, along with the results of classification and effectiveness are presented in Section 5. Section 6 concludes the paper and suggests topics for future work.

## 2. Related Works

Many studies concentrate on intracranial brain signals, in which electrodes are placed inside the skull directly on the brain. Antoniades et al. [12] used convolutional neural networks (CNN) applied with two convolutional layers on intracranial EEG data to extract the features of interictal epileptic discharge (IED) waveforms. The system divided the data into several 80 ms segments with 40 ms of overlap, and achieved a detection rate of 87.51%. 

Birjandtalab et al. [9] employed Fourier transform with deep neural networks (DNN) to classify the signals by applying the transform first on the obtained alpha, beta, gamma, delta, and theta as well as on the individual windows in order to calculate the power spectrum density that measures the signal power as a function of frequency. Then, DNN based on multilayer perceptrons with only two hidden layers was used to classify the signals. To avoid the overfitting problem, a few hidden layers were applied. The system achieved an accuracy of 95%. 

Seizure detection systems rely on the type of EEG data. Some of these systems detect epileptic seizures coming from only one channel, while others can detect epileptic seizures from multiple channels. ChannelAtt [13] is a novel channel-aware attention framework that adopts fully connected multi-view learning to soft-select critical views from multivariate bio signals. This model implements a new technique that relies on global attention in the view domain rather than the time domain. The system achieved a 96.61% accuracy rate.

Some studies performed feature learning by training the deep-learning model directly on EEG signals. Ihsan Ullah et al. [14] used a pyramidal 1D-CNN framework to reduce the amount of memory and the detection time. The final result used the voting approach for post-processing. To overcome the bottleneck of the requirement of training a huge amount of data, they performed data augmentation using overlapping windows. The system reached 99% accuracy.

Zabihi et al. [15] developed a system that combines non-linear dynamics (NLD) and linear discriminant analysis (LDA) for extracting the features and introduced the concept of nullclines to extract the discriminant features. The system employs artificial neural network (ANN) for classification. The yielded accuracy for the model was 95.11%. To mimic the real-world clinical situation, only 25% of the dataset was used for training. The results showed that the false negative rate was relatively high as a result of using a limited dataset for training. The sensitivity rates are considered too low for practical clinical use. 

Likewise, Avcu et al. [16] used a deep CNN algorithm on the EEG signals of 29 pediatric patients from KK Women’s and Children’s Hospital, Singapore. The researchers tried to minimize the number of channels in recorded EEG data to two channels only, Fp1 and Fp2. This data consists of 1037 min, of which only 25 min contain epileptic signals distributed over 120 seizure onsets. As seen, the data is not balanced. To overcome this problem, the researchers attempted to use various overlapping proportion techniques according to the seizures’ presence or absence by applying two shifting processes. The first one takes 5 s to create an interictal class (without overlapping). The second one takes 0.075 s to create an ictal class. These shifting processes were applied to balance the input data to the CNN. The system achieved an accuracy of 93.3%. However, the outcome of the data augmentation technique was not mentioned in this research.

Hu et al. [17] used long-short-term memory (LSTM) as it is efficient on both long-term and short-term dependencies in time series data. The authors developed the model using Bi-LSTM. The authors extracted and fed the network with seven linear features. The system was trained and tested on the Bonn University dataset, and it had a 98.56% accuracy. However, this reflects the accuracy of testing results, whereas the evaluation results were not mentioned in this research.

Chandel et al. [18] proposed a patient-specific algorithm that is based on wavelet-based features in order to detect onset-offset latency. The model operates by calculating statistical features such as mean, entropy, and energy over the wavelet sub-bands and then classifying the EEG signals using a linear classifier. The developed algorithm achieved an average accuracy of 98.60%. The algorithm was tested on 14 out of 23 patients in the dataset. Although the algorithm is patient-specific, its performance degraded significantly for patient 7, who had a very short seizure duration compared with the remaining patients; the number of seizures for this patient was 10, with a total duration of 94 s. This means that the algorithm performs well if the duration of the seizure is long, but falls significantly if the seizure is short.

Kaziha et al. [19] suggested using a model proposed in a previous study applied to the CHB-MIT dataset and tweaked to enhance performance. The model is based on five CNN layers, each of which is followed by a batch normalization and an average pooling layer, respectively. Finally, the model has three dense layers to detect the signal class. However, the performance chart of training and testing accuracy is an obvious indicator of the overfitting of a network, which can be seen from the sensitivity score. This is due to the imbalance of the dataset, as the number of epileptic signals is significantly lower than the number of non-epileptic signals, and therefore requires the use of a data augmentation scheme.

Huang et al. [20] suggested a three-part hybrid framework. The first part extracts the hand-crafted features and converts them into sparse categorical features, while the second part is based on a neural network architecture with the original signals as input to extract the deep features. Both types of extracted features are combined in the third and final part of the model for classifying the EEG signals into seizure and non-seizure. The model achieved a sensitivity score of 90.97%. It should be noted that the idea of the hybrid framework may achieve higher results if it enhances the output of the first part of the model, which are the features manually extracted from the signals. This is accomplished by using one of the feature-importance methods. A tree-based model is implemented to infer the importance score of each feature based on the decision rules (or ensembles of trees such as random forest) of the model.

Jeong et al. [21] implemented an attention-based deep-neural network to detect seizures. The model is divided into three modules; the first module extracts the spatial features, while the second module extracts the spatio-temporal features. The third module is the attention mechanism for capturing the representations that take into account the interactions among several variables at each point in time. The accuracy of the model is 89% and the sensitivity is 94%. However, based on the performance metrics of the model, the percentage of false negatives (FN), that is, the number of seizure signals that were detected as non-seizure, was low, which is reflected in the high sensitivity score. In contrast, the overall accuracy of the model was significantly lower compared with the sensitivity score, which means that the number of false positives (FP) was high. FP counts the number of non-seizure signals that were detected as seizures. Consequently, the model focused on extracting the features that would clearly distinguish the seizure class while not taking into consideration extracting the discriminative features for the non-seizure class as well. The overall performance of the model was affected. Table 2 summarizes all the above-mentioned studies in this section.

Most of the mentioned studies use augmentation to solve the issue of an imbalanced dataset. This research integrates two datasets using the intersection dominant channels between those datasets, followed by a deep-learning model to test the performance of the method.

## 3. Datasets

This section explains both the datasets that were used in the study. The first is the CHB-MIT dataset [11] that was collected from 22 subjects: 5 males aged 3–22 and 17 females aged 1.5–19. The dataset contains 969 h of EEG recordings, while the number of seizures is 198. The number of no-seizure signals exceeds the number of seizure signals. The second dataset is the KAU dataset that was collected from 2 male subjects aged 28 with scalp EEG recordings where the sampling frequency is the same as the CHB-MIT dataset, at 256 Hz. The age factor of the subjects was taken into consideration. The age of these two patients approximates the age of subjects in the CHB-MIT dataset. Hence, the range that was selected from both datasets was from 1–28. This is crucial as clinical and electroencephalographic characteristics of seizures depend greatly on age [22]. Both subjects have EEG recordings with 38 channels. One of them exhibited two seizures with a total duration of 495 s, while the other subject exhibited four seizures with a total duration of 417 s.

## 4. The Proposed System

This section is divided into two parts. The first part presents the compatibility framework, while the second part presents the seizure detection system.

### 4.1. Compatibility Framework for Data Integration

**Figure 1:**
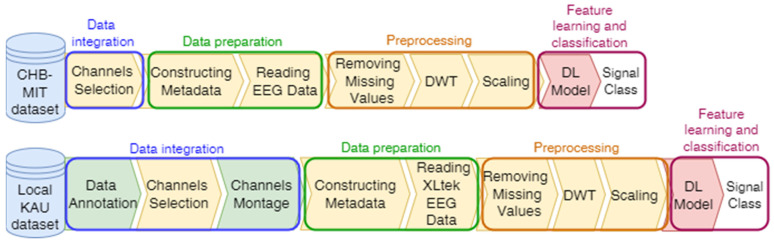
The proposed compatibility framework architecture.

The proposed system has a number of phases, including annotating the KAU dataset, selecting channels, and adjusting the channel montage, followed by a data preparation phase, which includes constructing metadata and reading EEG data. The third data preprocessing phase includes removing missing values, signal decomposition using the discrete wavelet transform (DWT), and scaling. Finally, the feature learning and classification phase, which is accomplished by a deep-learning (DL) model that classifies the EEG signals into seizure and non-seizure classes. Figure 1 illustrates the block diagram of the proposed system. The system is programmed by Colab, which is a Python development environment running on Google Cloud using the TensorFlow and Keras frameworks.

**Data Annotation of KAU Dataset:** The data were annotated in collaboration with the neurophysiologists and divided into categories: normal with open eyes, normal with closed eyes, pre-ictal, ictal, post-ictal, inter-ictal, and artifacts. Table 3 describes these categories.

**Figure 2:**
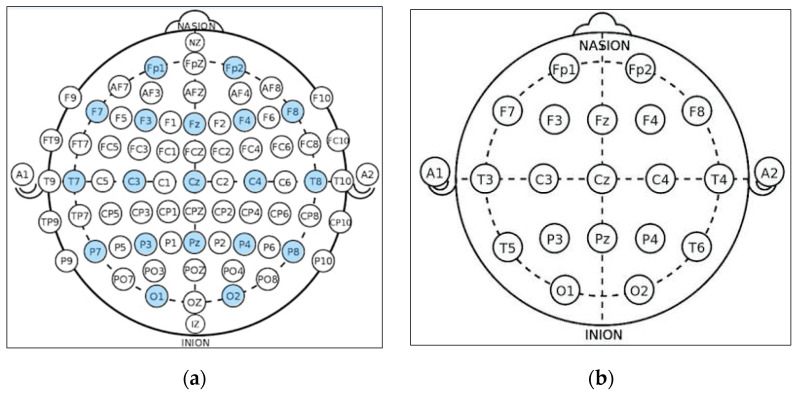
Schematic presentation of EEG electrode positions for: (**a**) CHB-MIT electrode positions where the adopted electrodes are highlighted with the blue color; (**b**) KAU electrode positions.

**Channels Selection:** In the CHB-MIT dataset, eighteen channels are selected out of twenty-three as these eighteen channels are the common channels among all the recordings. According to the distribution of electrode positions shown in Figure 2a, the adopted eighteen channels are: (‘C3-P3’, ‘C4-P4’, ‘CZ-PZ’, ‘F3-C3’, ‘F4-C4’, ‘F7-T7’, ‘F8-T8’, ‘FP1-F3’, ‘FP1-F7’, ‘FP2-F4’, ‘FP2-F8’, ‘FZ-CZ’, ‘P3-O1’, ‘P4-O2’, ‘P7-O1’, ‘P8-O2’, ‘T7-P7’, ‘T8-P8’). By comparing the KAU dataset with the CHB-MIT dataset in terms of the electrode positions, as shown in Figure 2, it is clear that the electrode locations in the two datasets are different. The majority of the electrodes in the CHB-MIT dataset are not present in the KAU dataset. Consequently, work was undertaken to replace the electrode that was not present with the nearest electrode in position as an alternative. The two datasets agree in the following electrodes: (‘C3-P3’, ‘C4-P4’, ‘Cz-Pz’, ‘F3-C3’, ‘F4-C4’, ‘FP1-F3’, ‘FP1-F7’, ‘FP2-F4’, ‘FP2-F8’, ‘Fz-Cz’, ‘P3-O1’, ‘P4-O2’). They differ in the rest of the electrodes. To demonstrate, the proposed system replaces the following electrodes: (‘F7-T7’ by ‘F7-T3’, ‘F8-T8’ by ‘F8-T4’, ‘P7-O1’ by ‘T5-O1’, ‘P8-O2’ by ‘T6-O2’, ‘T7-P7’ by ‘T3-T5’, ‘T8-P8’ by ‘T4-T6’).

**Channels Montage:** Montage refers to the arrangement of channels where the channel is a pair of electrodes. The KAU dataset channels are arranged in a common reference montage while the CHB-MIT dataset is bi-polar. The difference between these two types of montage is that the common reference montage compares the signal at every electrode position on the head to a single common reference electrode, whereas in the bi-polar montage, the signal consists of the difference between two adjacent electrodes [23]. To integrate both datasets, the proposed system changes the montage of the KAU dataset to the bipolar montage.

**Constructing Metadata:** The CSV files that contain the metadata are created for each patient. The metadata contains the file name, the recording start time, and the label given to the recording, where a label of 1 indicates seizure and a label of 0 indicates no-seizure. The EEG signal is divided for each seizure signal in each patient using a sliding window technique. This technique is a standard technique that has been adopted in other studies [24,25]. The sliding window technique with a fixed size was chosen to avoid the network parameter bias that may occur if the input signals to the network have a different length. The window size is *n* = 10 s with an overlap of k = 1 s. This technique was used in the incidence of a seizure EEG signal. In the case of the no-seizure EEG signal, there was no need for the overlapping. The CHB-MIT dataset constitutes about 24,000 windows of normal EEG records (no-seizure class) and about 434 windows of epilepsy EEG records (seizure class) for training data before the overlapping. It also constitutes about 6000 windows of normal EEG records (no-seizure class) and about 108 windows of EEG records (seizure class) for validation data prior to the overlapping. After the overlapping, the training data was about 24,000 windows for the no-seizure class and 4344 windows for the seizure class, whereas the validation data became 6000 windows for the no-seizure class and about 1086 windows for the seizure class. The window size was specifically chosen to be 10 s based on several factors. First, Table 4 shows the average duration of one seizure for some subjects in the dataset. It shows that subject 7 has a short average duration of a seizure compared with the remaining subjects in the dataset, as the minimum exposure time for seizures is 10 s on average depending on the dataset. Second, the model architecture is based on the use of the LSTM layer, with which the longer the window length, the more difficult the training becomes. To avoid data leakage, two points must be considered: (1) the dataset must be divided into training, validation, and testing sets before applying the overlapping technique; and (2) the overlapping technique must be applied to the data used for training only.

**Reading EEG Data:** The raw data and the metadata in CHB-MIT dataset are connected and analyzed using the wonambi library. The collected KAU dataset contains XLtek EEG data recorded using Natus Neuroworks. This type of EEG data consists of a set of files with different formats, comprised of: eeg, ent, epo, erd, etc, snc, stc, vt2, and vtc. The wonambi.ioeeg.ktlx module is used to ensure proper reading of the EEG signals. Algorithm 1 illustrates how to read XLtek EEG data. Note that the duration of each epoch in the proposed system is 10 s, comprising 46,080 samples.
**Algorithm 1.** **Reading XLtek EEG Data Algorithm.**
**Input**: An EEG signal and the size of window in seconds
**Output**: Array of EEG data samples that constitute the epochs**1****FUNCTION** get_epoch(s, min_secs = 10)**2** // Extracting signal start time, sample rate, channel names, and number of samples**3** start_time, s_rate, ch_names, n_samples ← s.return_hdr() **4** s_rate ← int(round(s_rate))**5** // Extracting the creation time for the erd file that holds the raw data**6** erd_time ← s.return_hdr() [−1][‘creation_time’]**7** // Excluding samples between the start time of recording and the actual acquisition**8** stc_erd_diff ← (erd_time–start_time). total_seconds()**9** // Computing the number of samples required from each channel**10** stride ← min_secs ∗ s_rate**11** start_index ← int(stc_erd_diff) ∗ s_rate**12** end_index ← start_index + stride**13** findings ← [ ]**14** **WHILE** end_index ≤ n_samples **DO****15**  t ← s.return_dat ([1], start_index, end_index)**16**  // Excluding the epochs that may contain NaN values**17**  **IF** ! np.any(np.isnan(t), axis = 1) **THEN****18**   data ← s.return_dat(range(len(ch_names)), start_index, end_index)**19**   **IF** s_rate > 256 **THEN****20**    data ← decimate(data, q = 2)**21**   **ENDIF****22**   // Converting numpy array to a pandas data frame**23**   df ← pd.DataFrame(data = data.T, columns = ch_names)**24**   findings.append(montage(df, model_modified_channels))**25**  **ENDIF****26**  start_index ← start_index + stride**27**  end_index ← end_index + stride**28** **ENDWHILE****29****return** findings**30****ENDFUNCTION**

**Removing Missing Values:** The Not-a-Number or NaN values were found and dropped in the proposed system because they were infrequent. 

**Wavelet Decomposition:** The proposed system utilizes a discrete wavelet transform (DWT) to decompose the signals. The signals are passed through high-pass and low-pass filters. The high-pass filter will generate all the high-frequency components, which are known as detailed coefficients. Similarly, the low-pass filter generates the wavelet coefficients, which are of low frequency and are known as approximation coefficients.

**Figure 3:**
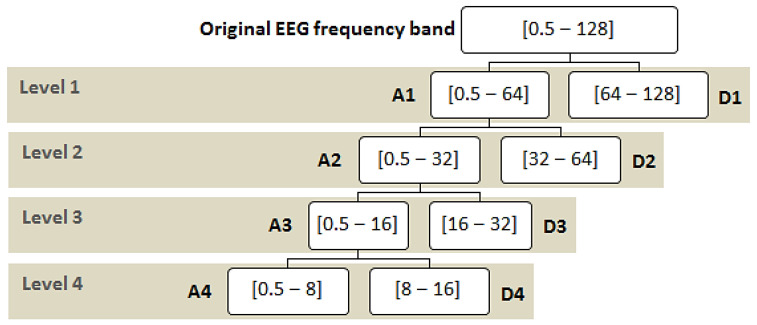
Proposed wavelet decomposition tree (db4).

The proposed system has a multi-level decomposition db4 which divides the wavelet into four levels. Each level represents a specific frequency band for the EEG signals that were previously referred to in Table 1, except for the first two frequency bands where the first DWT level in the proposed system represents both bands. Figure 3 shows the decomposition process of the original signal into two parts at the first level, where A1 refers to the approximation coefficients of the first level, while D1 refers to the detailed coefficients of the first level. The decomposition process continues after the first level until the fourth level in the same manner as the approximation coefficients only. The accepted coefficients in the proposed system from the DWT tree in Figure 3 are A4, D4, D3, and D2. A4 represents the delta and theta frequency bands, D4 represents the alpha frequency band, D3 represents the beta frequency band, and D2 represents the gamma frequency band. These accepted coefficients include the signals that are within the frequency range of 0.5 to 60 Hz because seizures are more distinguished in that range [26]. Furthermore, it ensures that many noises are removed, including power line noise, distinguished by a chronic sinusoidal component at 60 Hz that can be seen in raw biomedical data recordings. The sinusoidal element usually results from using devices that depend on alternating current as a power source [27].

**Figure 4:**
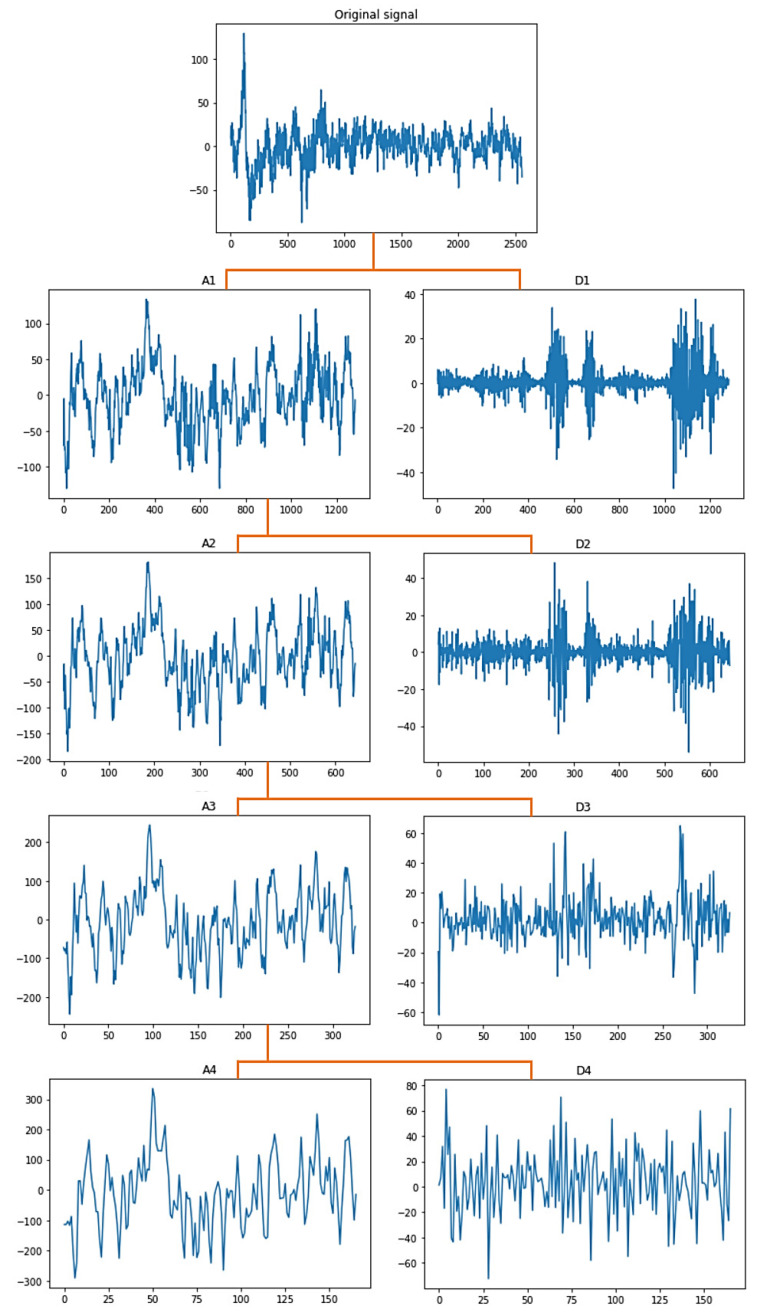
Approximation and detailed coefficients of the EEG signals.

Figure 4 shows the graphical representation of the EEG signal for each coefficient in the DWT tree shown in Figure 3. As seen after four decomposition levels, the width of the noisy signal (the approximation signal in the first level) is almost filtered compared with the last approximation signal in the last level because all high-frequency components at each level are taken out. So, the remaining approximation signal in the last level is a sine wave in filtered form.

**Scaling:** To speed up the model training process, the proposed model utilizes a scalar which is a z-score (standard score). The z-score is a statistical measurement which calculates the space between a data point and the mean [28]. In the proposed system, the z-score is performed on the batches. In this case, all the features will be transformed in such a way that they will have the properties of a standard normal distribution. In this scenario, the features will usually be in a bell curve. It was used because the model is based on deep-learning architecture, where it basically involves gradient descent, which in turn helps the TensorFlow and Keras libraries that are used when working with neural networks to learn the weights in a faster manner.

**Deep Learning Model**: A deep-learning model (DL model) that consists of several layers was used. In addition to these layers, auxiliary layers such as the activation and max-pooling 1D layers were used. The first helps in learning the non-linearity of the data, while the latter contributes to down-sampling the output of the convolutional layer (reducing dimensions) by selecting the maximum value on the filter.

**Figure 5:**
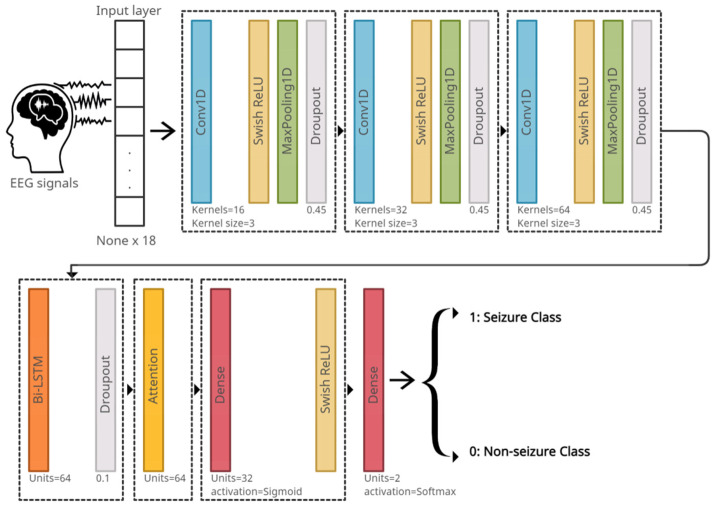
The deep-learning model architecture.

The DL model takes the EEG signals as an input. These signals are stored within one of the built-in data types in Python, which is a tuple. The dimensions of the tuple are (None ∗ 18), which indicates variable-length sequences of 18-dimensional vectors. It should be noted that the ‘None’ dimension means the network will be able to accept inputs from any dimension. Note that the window length is 10 s, the sample rate is 256, and the number of channels is 18. Therefore, the number of digital samples in each channel is 2560 samples, so the dimensions of any signal are (2560 ∗ 18), and after analyzing the signal using DWT, its dimensions will become (x ∗ 18), where x is the concatenation of the signal components after the decomposition procedure. Therefore, the dimensions of the signal become (A4 + D4 + D3 + D2, 18). In contrast, the model classifies these input EEG signals into two classes, seizure or non-seizure as an output. Figure 5 shows the order and the configurations of the layers in the model.

The loss function that is used in the proposed model is categorical cross-entropy. The adopted optimization algorithm for the model is the Adam algorithm [29]. One of the hyperparameters of the algorithm is the learning rate. The authors of Adam recommend setting the learning rate differently based on the system. It is better to use a decaying learning rate than a fixed one, which is a learning rate whose value decreases as the epoch number increases. This means it allows one to start with a relatively high learning rate while benefiting from lower learning rates in the final stages of training. This is useful where a relatively high learning rate is necessary to set huge steps, whereas increasingly smaller steps are necessary when approaching a minimum loss. The proposed model uses a learning rate with an initial value of 0.00001, taking into account the use of a common decay scheme, which allows learning rates to be dropped in smaller steps exponentially every few epochs.

### 4.2. Seizure Detection Model

The proposed system is trained, validated, and tested on the CHB-MIT Scalp EEG dataset. It depends on the eighteen common channels that have been previously mentioned. The model suggested in Figure 5 is used, except each dropout layer is replaced by a batch normalization layer. The EEG signals are inputted to the system and passed through three CNN layers, each with different configurations as shown in Figure 5. Next are the Bi-LSTM and attention layers, respectively. Finally, the signals pass through two dense layers that classify the signal as seizure or non-seizure.

**Convolutional Neural Network:** The EEG signals are one-dimensional time series data; hence, for its analysis, a one-dimensional CNN is proposed (1D-CNN). The 1-D CNN automatically learns the discriminative features that represent the structure of EEG signals [30]. 

The activation function for the proposed model is the Swish Rectified Linear Unit (Swish Relu) [31]. The activation function’s purpose is to classify and learn the non-linearity in the data. The formula for Swish Relu is as follows:

f(x) = x ∗ sigmoid(βx)(1)

where:

sigmoid(βx) = 1/(1 + e (−βx)(2)

where β is a constant; if β is close to 0, the function will work linearly. If β is a large value, greater than or equal to 10, the function works similarly to Relu. After performing some experimental work, it is considered β = 1 in this study.

**Max Pooling:** Max-pooling 1D [32] is an operation which is usually appended to CNNs after the individual convolutional layers to down-sample the output. Max pooling is applied to reduce the resolution of the output of the convolutional layer, which decreases the network parameters and subsequently decreases the computational load as well as the overfitting. It is also helpful in selecting the higher valued frequencies as being the most activated frequencies. The filter (window) of size 3 is applied in the proposed system.

**Batch Normalization:** Throughout training, the distribution of the input data varies due to the update of the parameters. This will slow down the learning, so the learning becomes harder with nonlinearities. This phenomenon is called internal covariate shift [33]. To solve this issue, batch normalization is used. This makes the optimization significantly smoother, speeds up the training process, and slightly regularizes the model.

**Bidirectional Long Short-Term Memory:** Bidirectional LSTM (Bi-LSTM) [34] divides the standard LSTM’s hidden neuron layer into two propagation directions: forward and backward. Therefore, this structure of Bi-LSTM will make it capable of processing the input in two ways: modeling from the front to the back and from the back to the front. The Bi-LSTM has the ability to detect the contextual information in long sequences of data and learn the importance of different events. For this purpose, the proposed system uses Bi-LSTM. In fact, the Bi-LSTM in the proposed model will make full use of the information before and after the states of epileptic seizure, enabling seizure events to be properly detected. The number of units of Bi-LSTM represents the dimensionality of the output space.

**Attention:** Attention [35] is the ability to highlight and use the salient parts of information dynamically in a similar way to the human brain. This type of mechanism works through iterative re-weighting to allow the model to utilize the most relevant components of the input sequence, which is the EEG signal, in a flexible manner in order to give these relevant components the highest weights. This type of mechanism was initially proposed and is usually used to process sequences such as EEG signals. For this reason, it was used in the proposed model. The Bi-LSTM with attention is a way to significantly enhance the model performance.

**Fully Connected Layer:** The fully connected layer [36] works as a classifier and predicts the input signal class. The proposed system has two dense layers. The first layer consists of thirty-two units (neurons), which represent the dimensionality of the output space. The second dense layer in the model has two units because the proposed model classifies the EEG signals into two classes: seizure or non-seizure. The reason for using two dense layers instead of one is that the convolution layers, in conjunction with the Bi-LSTM and attention layers, extract the features from the EEG signals. Depending on these features, the deep-neural network layers classify the signals. The first dense layer acts as a feature selector to decide whether or not a feature is relevant to a class, whereas the second dense layer acts as a classifier. Thus, the presence of two dense layers enhances the network’s ability to better classify the extracted features.

## 5. The Experimental Result

This section will be divided into two parts. The first one is to evaluate the compatibility framework for integrating local EEG data with the CHB-MIT dataset. The second one is to evaluate the seizure detection model.

### 5.1. Evaluating the Compatibility Framework

To assess the possibility of data integration, the DL model uses a set of well-known performance metrics to measure the model’s performance: sensitivity, precision, and accuracy. The formulas for these metrics are shown below:

*Sensitivity (Recall or Sen.) = TP/(FN + TP)* (3)

*Precision (PRC) = TP/(TP + FP)* (4)

*Accuracy (ACC) = (TP + TN)/(Total Samples)* (5)

where *TP* (True Positive) is the number of seizure signals that are detected as seizure, *FN* (False Negative) is the number of seizure signals that are detected as non-seizure, *TN* (True Negative) is the number of non-seizure signals that are detected as non-seizure, and *FP* (False Positive) is the number of non-seizure signals that are detected as seizure. 

A set of experiments were performed to demonstrate the feasibility and usefulness of the deep-learning model for proving the concept of data integration and effectiveness of the compatibility framework with CHB-MIT dataset standards.

Initially, a random sample of EEG signals was taken from the CHB-MIT dataset for each experiment. Considering that the number of random EEG signals in the sample is proportional to the number of EEG signals extracted from the KAU dataset, the impact of KAU EEG signals can be studied by integrating them with the random sample. To clarify, the number of EEG signals extracted from the KAU dataset was 185 signals for both classes, and the number of random EEG signals in each sample was 750 signals. Therefore, the number of EEG signals from the KAU dataset constituted approximately 25% of the random sample size, which allows measuring the effectiveness of data integration. To illustrate, the number of EEG signals in each random sample from the CHB-MIT dataset was proportional to the number of EEG signals extracted from the KAU dataset in order to ensure that the impact of data integration from the KAU dataset with the CHB-MIT dataset was studied. The selection of signals in the sample was random to ensure that the effect of integration was properly studied. Therefore, multiple experiments were conducted with multiple random samples.

Six different experiments were performed as displayed in Table 5. Each experiment aims to measure the DL model performance on the sample extracted from the CHB-MIT dataset, and to merge the KAU EEG signals with a random sample also from the CHB-MIT dataset to study the effect of the data that is attached to the CHB-MIT dataset. 

For further illustration, each random sample taken from the CHB-MIT dataset contained 750 random signals, which were then divided into training, validation, and testing at 50%, 20%, and 30%, respectively, so that the number of training signals was 375 and the number of testing signals was 225. It should be noted that the number of seizure signals was equal to the number of non-seizure signals in the first three experiments carried out on the CHB-MIT dataset only. The KAU EEG datasets were then randomly subdivided into training, validation, and testing groups. After that, these samples from KAU EEG data were merged with three random samples from the CHB-MIT dataset. 

As noted in Table 5, the values of the performance metrics for each experiment before and after merging the random sample with the KAU EEG data are enhanced or within the same range, proving that the integration of data with the KAU dataset using the proposed framework is effective to combat the problem of data imbalance.

As seen, the proposed compatibility framework for creating a large and balanced dataset by integrating the EEG signals from the KAU dataset with the CHB-MIT dataset showed an improvement in the ability of the model to identify seizure signals with higher accuracy. The system suggested increasing the number of epilepsy signals and measuring the impact of integration on the performance of the model in terms of the overall accuracy of detecting epileptic seizures before and after the integration process. The overall accuracy of 78.85% increased to 80.15%. In particular, the performance improved through the sensitivity rate to epileptic seizures specifically; it was initially 62.51% and became 68.13%, meaning that the number of seizure signals that were detected as non-seizure was low, as reflected in the high sensitivity rate.

**Figure 6:**
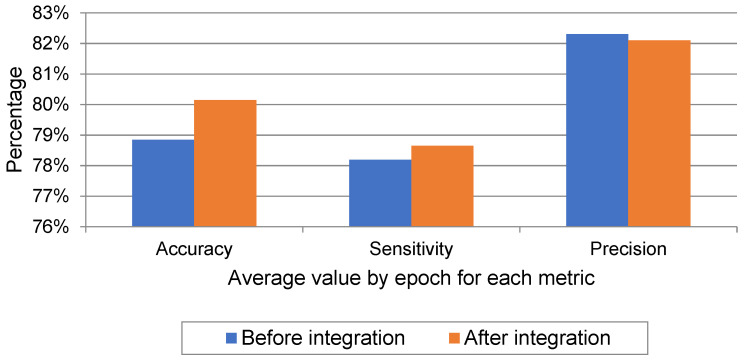
Average values of experiments before and after data integration for performance metrics.

The model was trained on Google Colab using an Nvidia Tesla K80 GPU. Figure 6 shows the average values by epoch of the metrics that were previously mentioned in Table 5 for both classes of seizure and no-seizure. Through it, we note the high level of sensitivity after data integration which measures the percentage of seizure signals that were classified as seizure. However, we also observe from the chart that the level of precision slightly decreased after data integration which measures the proportion of no-seizure signals that were classified as no-seizure. The reason for this is the presence of artifact signals in the KAU dataset, which in turn were classified as seizure signals. This problem can be solved in future work by incorporating a tool into the model that deals with artifact signals. Finally, we notice an increase in overall accuracy after the data integration process, despite the decrease in precision, and the reason for this is the high sensitivity.

### 5.2. Evaluating the Seizure Detection Model

For evaluation and testing, 20% and 30% of the CHB-MIT dataset were used, respectively. The testing data constitutes about 12,000 windows of normal EEG records (no-seizure class) and about 3004 windows of epilepsy EEG records (seizure class). The performance was evaluated using the same performance metrics that are used to evaluate the compatibility framework, which are sensitivity, precision, and accuracy. 

A comparison of the proposed model with state-of-the-art methods trained and tested on CHB-MIT is given in Table 6. As seen, the proposed system outperforms the previous systems, except for one [18] study. However, when we compare the proposed system with that study, we find that the study was only tested on 14 of the 23 patients in the dataset, but the proposed system was evaluated on all 23 patients. In addition, we find that although the algorithm for that study is patient-specific, its performance deteriorated significantly for patient 7, where the sensitivity rate reached 50%, because the duration of epileptic seizures for this patient was very short. This means that the algorithm works well if the duration of the seizure is long. However, if the seizure is brief, the accuracy drops dramatically. The proposed system provides good performance in both cases, whether the duration of the seizure is long or short, as seen through the sensitivity ratio of the proposed system, which was tested on all patients and overcame the sensitivity of the previous model.

The uniqueness of the proposed deep-learning model lies in its design topology that suggests specific types of layers with specific configuration parameters, as in Figure 5, where the configuration of this model makes it capable of outperforming state-of-the-art models by combining several advantages in the network design. First, it visually extracts the signal abnormalities from the 1D-EEG through the Conv1D, which is a visual neural network. Second, it learns the non-linearity in the EEG signals through swish Relu. Third, it identifies some distinct features from the higher valued frequencies as being the most activated frequencies through max-pooling. Fourth, it learns the seizure and no-seizure events from the contextual information before and after the states of epileptic or non-epileptic signals in forward and backward propagation directions through Bi-LSTM. Fifth, it improves the performance of the model significantly by combining attention with Bi-LSTM to give the relevant components the highest weights during the iterative re-weighting process.

Since the EEG patterns are highly subject-dependent, the main contribution of the proposed model is to deal with dual-detection problems (seizure versus non-seizure) based on using a small number of channels that are common for all patients, not for each patient separately, to achieve better performances than those of systems of full channels.

A limitation of the proposed model could be the inability to detect the seizure or no-seizure from the EEG signals with a sample rate of 512 Hz. For further improvement, the model can be trained using the decimate() method to down-sample the signal that has a sample rate of 512 Hz, which would enable the model to detect epileptic seizures from signals with a sampling rate of 256 or 512 Hz.

**Figure 7:**
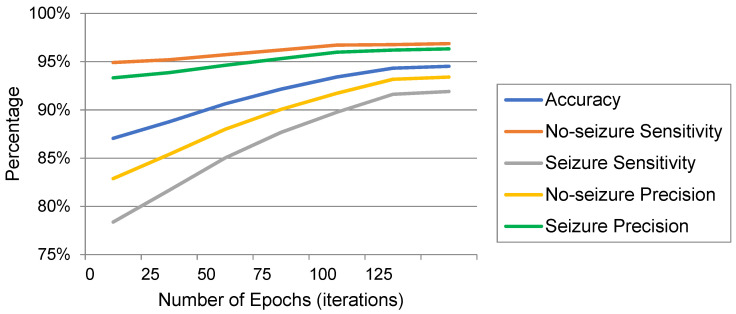
The performance metric charts of testing against the epochs.

The model was trained on Google Colab using an Nvidia Tesla K80 GPU. Figure 7 shows the performance of the model by epoch for testing according to the metrics that were previously used in Table 6 for each class, seizure or no-seizure. We observe that the convergence of the model occurred at the 130th epoch. Comparing Kaziha et al. [19] with our model, our method shows a better sensitivity of 96.85% while theirs was 82.35%. One of the main reasons is that their window size was 100 s, whereas our window size was 10 s, which in turn takes only the exact seizure intervals.

## 6. Conclusions

In this research, a compatibility framework for integrating local EEG signals into the CHB-MIT dataset is proposed. The proposed approach has multiple benefits. First, it overcomes the problem of data imbalance faced by most of the datasets in the field due to the low incidence of epileptic signals compared to non-epileptic signals. Second, it allows the establishment of large datasets by integrating local EEG signals with the available datasets required by the deep-learning models used to develop seizure detection and prediction systems. The approach presented in this paper can also be used as a support tool for researchers in the field to process and read local EEG signals that are of the XLtek type for which there were no reading functions available in the analysis software packages for such EEG types. In the end, a set of experiments carried out to examine the data integration using the proposed framework proved its feasibility and usefulness. 

In addition, an automated epilepsy detection system that is based on some channels was proposed. This system deals with dual-detection problems (seizure versus non-seizure). The proposed system uses a wavelet decomposition technique and a simple one-dimensional convolutional neural network, along with bidirectional long-short-term memory and attention, to receive EEG signals as input data, pass them to various layers, and finally make a decision via a dense layer. This model can assist neurophysiologists to detect the seizures and significantly decrease the burden, while also increasing the efficiency. 

There are several future suggestions regarding the proposed model. One such suggestion is that it could be incorporated into a wearable device for patients, considering the storage and memory requirements. Another suggestion is the possibility of deploying the system in a central cloud environment for rapid access via mobile devices without using specific wear-and-tear devices. The EEG signal that is considered as the input data is small in size and the proposed model is portable, which makes it appropriate for cloud deployment. The EEG signals are easily transferred to the cloud for processing in real-time as it can issue a warning alarm to notify the doctors/patients if needed. The proposed system can be used to implement expert systems for similar disorders that include EEG brain signals.

## Figures and Tables

**Table 1:** The frequency bands of EEG signals [8].

Frequency	Bandwidth	Normal Tasks	Abnormal Tasks
0.1–4 Hz	Delta (δ)	sleep, artifacts, hyperventilation	structural lesion, seizures, encephalopathy
4–8 Hz	Theta (θ)	drowsiness, idling	encephalopathy
8–12 Hz	Alpha (α)	closing the eyes, inhabitation	coma, seizures
12–30 Hz	Beta (β)	effect of medication, drowsiness	drug overdose, seizures
30–70 Hz	Gamma (γ)	voluntary motor movement, learning and memory	seizures

**Table 2:** EEG-based epileptic seizure detection systems using deep-learning approaches.

Cite	Published Year	Approach	Layers	Dataset	Channels	Accuracy	Window Size
[12]	2016	CNN	2	King’s College London Hospital dataset	12 channels	87.51%	80 ms
[9]	2017	Deep Neural Networks	4	23 epileptic patients from Boston Children’s Hospital	Ranges from 18 to 23 channels	95%	10 s
[13]	2018	Channel-aware Attention Framework	23	CHB-MIT dataset	23 channels (in few cases 24 or 26)	96.61%	NA
[14]	2018	Pyramidal one-dimensional CNN models	3	Bonn university dataset	1 channel	99%	10 s
[15]	2019	Nonlinear dynamics (NLD) with Linear Discriminant Analysis (LDA) and Artificial Neural Network (ANN)	5	CHB-MIT dataset	23	95.11%	1 s
[16]	2019	Deep CNN	4	29 pediatric patients from KK Women’s and Children’s Hospital, Singapore	2 channels	93.3%	5 s
[17]	2019	Deep Bi-LSTM Network	5	Bonn university dataset	1 channel	98.56%	NA
[18]	2019	Discrete Wavelet Transform (DWT) + linear classifier	NA	CHB-MIT dataset	23 channels (in few cases 24 or 26)	98.60%	1 s
[19]	2020	CNN	18	CHB-MIT dataset	23 channels (in few cases 24 or 26)	96.74%	100 s
[20]	2021	Gradient-Boosted Decision Trees (GBDT) with Deep Neural Network (DNN)	NA	CHB-MIT dataset	23 channels (in few cases 24 or 26)	NA	20 s
[21]	2021	CNN	20	CHB-MIT dataset	23 channels (in few cases 24 or 26)	89%	NA

**Table 3:** Description Of EEG Categories For Annotated Local Dataset.

Category	Description
Open eyes	EEG recording for a relaxed patient in awake state with eyes open
Closed eyes	EEG recording of a relaxed or sleeping patient with eyes closed
Pre-ictal	EEG recording for a patient in a state prior to epileptic seizure
Ictal	EEG recording for a patient during epileptic seizures
Post-ictal	EEG recording for a patient in a state posterior to epileptic seizure
Inter-ictal	EEG recording for a patient in seizure-free interval between seizures
Artifacts	Signals recorded by EEG that might mimic seizures but generated from outside the brain

**Table 4:** Seizure duration for a sample of subjects in the CHB-MIT dataset.

Subject No.	Total Number of Seizures	Total Seizures Duration (Seconds)	Average Seizure Duration (Seconds)
1	7	449	64.14
3	7	409	58.43
5	4	280	70
7	10	94	9.4
9	6	323	53.83

**Table 5:** The performance of the DL model with and without data integration.

EXP No.	DB	Avg. Epoch ACC	Avg. Epoch Sen. for Seizure	Avg. Epoch Sen. for No-Seizure	Avg. Epoch PRC for Seizure	Avg. Epoch PRC for No-Seizure
**1**	CHB-MIT	79.25	64.16	93.14	89.2	75.29
**2**	CHB-MIT	81.93	68.43	94.41	91.54	78.03
**3**	CHB-MIT	75.38	54.95	94.02	89.26	70.53
**Avg.**	**CHB-MIT**	78.85	62.51	93.86	90	74.62
**4**	CHB-MIT + KAU	77.81	66.76	88.01	84.01	76.99
**5**	CHB-MIT + KAU	80.90	75.34	84.66	78.09	86.03
**6**	CHB-MIT + KAU	81.73	62.29	94.8	87.71	79.78
**Avg.**	**CHB-MIT + KAU**	80.15	68.13	89.16	83.27	80.93

**Table 6:** Performance comparison of the proposed model with other systems on the CHB-MIT dataset.

Cite	No. of Channels	No. of Subjects	Sen.	PRC	ACC	Speed of Convergence
[13]	23 channels (in few cases 24 or 26)	23	-	96.51	96.61	NA
[15]	23	25% of the dataset	91.15	-	95.11	NA
[18]	23	14 specific patients	96.43	-	98.60	NA
[19]	23 channels (in few cases 24 or 26)	23	82.35	-	96.74	Around 60 epochs
[21]	23 channels (in few cases 24 or 26)	23	90.97	-	-	NA
[20]	23 channels (in few cases 24 or 26)	23	94	-	89	NA
**The proposed model**	18 channel	23	96.85	96.98	96.87	Around 130 epochs

## Data Availability

The CHB-MIT datasets analyzed during the current study are available in the PhysioNet repository [https://physionet.org/content/chbmit/1.0.0/ accessed on 10 August 2020]. While the KAU datasets that support part of the findings of this study are available from King Abdulaziz University Hospital, restrictions apply to the availability of these data, which were used under license for the current study, and so are not publicly available. Data is, however, available from the authors upon reasonable request and with permission of King Abdulaziz University Hospital.

## References

[1] Panayiotopoulos C. (2010). A Clinical Guide to Epileptic Syndromes and Their Treatment.

[2] World Health Organization (2018). Epilepsy. http://www.who.int/en/news-room/fact-sheets/detail/epilepsy.

[3] (2018). Background to Seizures. Epilepsy Research UK. https://www.epilepsyresearch.org.uk/about-epilepsy/background-to-seizures/.

[4] Bell G., Sinha S., Tisi J., Stephani C., Scott C., Harkness W., McEvoy A., Peacock J., Walker M., Smith S. (2010). Premature mortality in refractory partial epilepsy: Does surgical treatment make a difference?. J. Neurol. Neurosurg. Psychiatry.

[5] Ulate-Campos A., Coughlin F., Gaínza-Lein M., Fernández I., Pearl P., Loddenkemper T. (2016). Automated seizure detection systems and their effectiveness for each type of seizure. Seizure.

[6] (2022). EEG (Electroencephalogram)—Mayo Clinic. https://www.mayoclinic.org/tests-procedures/eeg/about/pac-20393875.

[7] Nacy S., Kbah S., Jafer H., Al-Shaalan I. (2016). Controlling a Servo Motor Using EEG Signals from the Primary Motor Cortex. Am. J. Biomed. Eng..

[8] Tatum W.O. (2014). Ellen R. grass lecture: Extraordinary EEG. Neurodiagnostic J..

[9] Birjandtalab J., Heydarzadeh M., Nourani M. Automated EEGbased epileptic seizure detection using deep neural networks. Proceedings of the 2017 IEEE International Conference on Healthcare Informatics (ICHI).

[10] Buda M., Maki A., Mazurowski M.A. (2018). A systematic study of the class imbalance problem in convolutional neural networks. Neural Netw..

[11] Shoeb A. (2009). Application of Machine Learning to Epileptic Seizure Onset Detection and Treatment. Ph.D. Thesis.

[12] Antoniades A., Spyrou L., Took C.C., Sanei S. Deep learning for epileptic intracranial EEG data. Proceedings of the 2016 IEEE 26th International Workshop on Machine Learning for Signal Processing (MLSP).

[13] Yuan Y., Xun G., Ma F., Suo Q., Xue H., Jia K., Zhang A. A novel channel-aware attention framework for multi-channel EEG seizure detection via multi-viewdeep learning. Proceedings of the 2018 IEEE EMBS International Conference on Biomedical & Health Informatics (BHI).

[14] Ullah I., Hussain M., Qazi E.-U.-H., Aboalsamh H. (2018). An automated system for epilepsy detection using EEG brain signals based on deep learning approach. Expert Syst. Appl..

[15] Zabihi M., Kiranyaz S., Jantti V., Lipping T., Gabbouj M. (2019). Patient-Specific Seizure Detection Using Nonlinear Dynamics and Nullclines. IEEE J. Biomed. Health Inform..

[16] Avcu M.T., Zhang Z., Chan D.W.S. Seizure detection using least EEG channels by deep convolutional neural network. Proceedings of the ICASSP 2019 IEEE International Conference on Acoustics, Speech and Signal Processing (ICASSP).

[17] Hu X., Yuan Q. Epileptic EEG Identification Based on Deep Bi-LSTM Network. Proceedings of the 2019 IEEE 11th International Conference on Advanced Infocomm Technology (ICAIT).

[18] Chandel G., Farooq O., Khan Y., Varshney Y. Patient Specific Seizure Onset-Offset Latency Detection using Long- term EEG Signals. In Proceedings of the 2019 International Conference on Electrical, Electronics and Computer Engineering (UPCON).

[19] Kaziha O., Bonny T. A Convolutional Neural Network for Seizure Detection. Proceedings of the 2020 Advances in Science and Engineering Technology International Conferences (ASET).

[20] Huang C., Chen W., Chen M., Yuan B. (2021). A Feature Fusion Framework and Its Application to Automatic Seizure Detection. IEEE Signal Process. Lett..

[21] Jeong S., Jeon E., Ko W., Suk H. Fine-grained Temporal Attention Network for EEG-based Seizure Detection. In Proceedings of the 2021 9th International Winter Conference on Brain-Computer Interface (BCI).

[22] Holmes G. (2012). Consequences of Epilepsy through the Ages: When is the Die Cast?. Epilepsy Curr..

[23] Jadeja N.M. (2021). Montages. How to Read an EEG.

[24] Sharmila A., Geethanjali P. DWT Based Detection of Epileptic Seizure From EEG Signals Using Naive Bayes and k-NN Classifiers. Proceedings of the 2017 International Conference on Trends in Electronics and Informatics (ICEI).

[25] Zhang T., Chen W., Li M. (2017). AR based quadratic feature extraction in the VMD domain for the automated seizure detection of EEG using random forest classifier. Biomed. Signal Process. Control.

[26] Khan Y.U., Farooq O., Sharma P. (2012). Automatic detection of seizure onset in pediatric EEG. Int. J. Embed. Syst. Appl..

[27] Akwei-Sekyere S. (2015). Powerline noise elimination in biomedical signals via blind source separation and wavelet analysis. PeerJ.

[28] Frost J. (2022). Z-score: Definition, Formula, and Uses. Statistics by Jim. https://statisticsbyjim.com/basics/z-score/.

[29] Kingma P.D., Ba J.L. (2017). Adam: A method for stochastic optimization. arXiv.

[30] Albawi S., Mohammed T.A., Al-Zawi S. Understanding of a convolutional neural network. Proceedings of the 2017 International Conference on Engineering and Technology (ICET).

[31] Ramachandran P., Zoph B., Le Q.V. (2017). Searching for activation functions. arXiv.

[32] Murray N., Perronnin F. Generalized Max Pooling. Proceedings of the 2014 IEEE Conference on Computer Vision and Pattern Recognition.

[33] Ioffe S., Szegedy C. (2015). Batch normalization: Accelerating deep network training by reducing internal covariate shift. arXiv.

[34] Aggarwal R. (2019). Bi-LSTM. Medium.

[35] Verma Y. (2022). A Beginner’s Guide to Using Attention Layer in Neural Networks. Analytics India Magazine.

[36] Unzueta D. (2021). Convolutional Layers vs. Fully Connected Layers. Towards Data Science. https://towardsdatascience.com/convolutional-layers-vs-fully-connected-layers-364f05ab460b.

